# Quantum Dot-Based Lateral Flow Immunoassay as Point-of-Care Testing for Infectious Diseases: A Narrative Review of Its Principle and Performance

**DOI:** 10.3390/diagnostics12092158

**Published:** 2022-09-06

**Authors:** Mohamad Ahmad Najib, Kasturi Selvam, Muhammad Fazli Khalid, Mehmet Ozsoz, Ismail Aziah

**Affiliations:** 1 Institute for Research in Molecular Medicine (INFORMM), Health Campus, Universiti Sains Malaysia, Kubang Kerian 16150, Kelantan, Malaysia; 2 Department of Biomedical Engineering, Near East University, via Mersin 10, Nicosia 99138, Turkey

**Keywords:** quantum dot, lateral flow immunoassay, infectious diseases

## Abstract

Infectious diseases are the world’s greatest killers, accounting for millions of deaths worldwide annually, especially in low-income countries. As the risk of emerging infectious diseases is increasing, it is critical to rapidly diagnose infections in the early stages and prevent further transmission. However, current detection strategies are time-consuming and have exhibited low sensitivity. Numerous studies revealed the advantages of point-of-care testing, such as those which are rapid, user-friendly and have high sensitivity and specificity, and can be performed at a patient’s bedside. The Lateral Flow Immunoassay (LFIA) is the most popular diagnostic assay that fulfills the POCT standards. However, conventional AuNPs-LFIAs are moderately sensitive, meaning that rapid detection remains a challenge. Here, we review quantum dot (QDs)-based LFIA for highly sensitive rapid diagnosis of infectious diseases. We briefly describe the principles of LFIA, strategies for applying QDs to enhance sensitivity, and the published performance of the QD-LFIA tested against several infectious diseases.

## 1. Introduction

Infectious diseases have become more persistent in recent decades amid advances in medical science [1]. Diseases such as COVID-19, typhoid fever, tuberculosis, cholera, malaria, measles, hepatitis, Ebola virus disease, acquired immunodeficiency syndrome (AIDS), and influenza viruses are a serious threat to human health, as well as having serious negative impacts on social life and global economics [2]. The main drivers of emerging infectious disease outbreaks are attributed to human activities that lead to environmental changes, increased international mobility due to advances in transportation and logistics, and microbial adaptations [3,4]. To combat infectious diseases effectively, scientific communities utilise a variety of approaches focusing on the rapid detection and surveillance of pathogens with the potential to cause outbreaks, epidemics, and even pandemics.

Point-of-care testing (POCT) technology has exhibited an outstanding capability for the detection of several disease biomarkers owing to the fact that such techniques are fast, easy to perform, efficient, and low cost. POCT enables health practitioners to make faster clinical decisions so that appropriate treatment can be implemented. In addition, POCT is an important diagnostic tool for controlling disease outbreaks, especially in low-resource settings [5,6]. With the advantages of its low cost, POCT has been widely used in mass screening surveillance programs to prevent the spread of COVID-19. Several new strategies for POCT diagnostic tools have been developed in recent years [7,8,9]. The lateral flow immunoassay (LFIA) is one such strategy for POCT. LFIA is a well-established platform and a potent assay for fast and inexpensive testing, as this technology is instrumentation independent and allows the visualisation of test results by the naked eye. All LFIAs utilise reagents stored in both dry and liquid forms [10]. In the early 1980s, the first commercial LFIA was marketed to detect human chorionic gonadotropin (hCG). Since then, the LFIA methodology has garnered the attention of researchers from a wide range of fields. This is mainly due to LFIAs readily fulfilling the ASSURED (Affordable, Sensitive, Specific, User-friendly, Rapid/Robust, Equipment-free, and Deliverable to end users) criteria for POCT devices as recommended by the World Health Organization (WHO). In addition, a large plethora of body fluid could be reduced by utilising POCT compared to the gold standard (culture method) and molecular method (PCR), suggesting the powerful feature of POC devices. Currently, a wide range of LFIAs are now available for the detection of various biomarkers of communicable and non-communicable diseases, as well as toxins in food samples [11].

Conventional LFIAs utilise nanomaterials such as gold nanoparticles (AuNPs) as a reporter, allowing results visualisation with the naked eye. However, most LFIAs functionalised with AuNPs are moderately sensitive and only provide qualitative information [12]. As an alternative to AuNPs, various reporters have been used in LFIAs, such as latex microparticles and europium nanoparticles [13]. Latex microparticles derived from polymer (commonly polystyrene) are used in LFIAs due to their unique colour flexibility. The latex microparticles have the advantage of optical properties, which are suitable for the development of multiplex LFIAs [14]. On the other hand, europium nanoparticles have been used in LFIAs to detect common food contaminants, such as the antibiotic residues of veterinary drugs [15]. The LFIA showed a high sensitivity for the detection of tetracyclines, sulfonamides, and fluoroquinolones. A report has shown that europium causes human safety issues such as respiratory tract, skin, and eye irritation. Furthermore, the synthesis method of these nanoparticles has some disadvantages, in that the obtained nanoparticles were normally not well dispersed, and discarding assays is not environmentally safe [13].

In order to enhance the sensitivity and specificity of the LFIAs, as well as to allow the quantitation of results, fluorescence immunochromatographic assays have been developed by utilising fluorescent reporters [16,17,18,19,20]. Fluorescence immunochromatographic assays have advantages over conventional approaches in regard to sensitivity as it produces a higher intensity band on the test and control lines [21,22]. One such promising fluorescent reporter is quantum dots (QDs). QDs are tiny semiconducting nanocrystals with diameters ranging from 2 to 10 nanometers. QDs have unique electronic characteristics that are intermediate between those of bulk semiconductors and discrete molecules, which is due in part to their high surface-to-volume ratios. The most visible result is fluorescence, in which the nanocrystals emit distinct colours determined by particle size. Hence, the present review focuses on a quantum dot-based lateral flow immunoassay (QD-LFIA). Specifically, we start with the main principle of the lateral flow immunoassay and conventional reporter used for signal measurement. Then, we describe the two main formats of the LFIA. Finally, we describe the types of QDs, principles of QD-LFIA, covalent linkage chemistry for conjugating QDs with antibodies, and the published performance of QD-LFIAs in terms of the limit of detection, as well as the strategies utilised by researchers.

## 2. Lateral Flow Immunoassay

Lateral flow immunoassay (LFIA) is an immunochromatographic paper-based assay for detecting targets in complex mixtures [23]. LFIAs have several key features. First, the assays are rapid, where the visualisation of the test results can be performed in less than 30 min. Second, the assays can automatically separate the target analytes from the biological samples without sophisticated extra steps such as washing steps in enzyme-linked immunosorbent assays (ELISA). The assay is usually based on antigen–antibody interaction, and sample movement across the membrane occurs via capillary force [24]. Finally, the assays can be operated without the need for expensive equipment and highly trained staff to perform sophisticated analytical procedures, making them suitable for POCT and field-based diagnostic uses. Since the LFIA is an antibody-based approach, other compounds with similar structures may impact its specificity and sensitivity, producing false positive results. In addition, the Kd (dissociation constant) of the antibody–antigen and kinetic rate constant are especially important since LFIA is an in-flow system where the antigen–nanoparticle complex is in contact with the capture antibody for a limited time. The Kd of antibody–antigen conjugate and the colourimetric readout serve as constraints on test sensitivity. Readers and unique biochemical approaches have been developed to enhance product quality and customer comfort in order to overcome these constraints.

**Figure 1:**
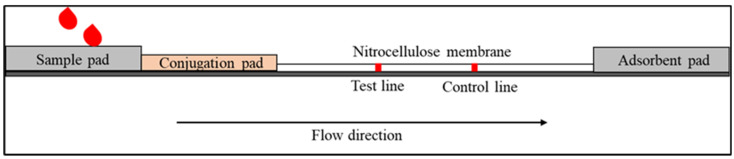
Schematic diagram shows the basic structure of LFI that consists of a sample pad, conjugation pad, nitrocellulose membrane and adsorbent pad.

The standard LFIA device comprises four components [25]. The sample pad is the first component for sample loading. The sample pad serves to absorb the sample and control the distribution of the sample to the second part. In addition, the sample pad also acts as a filter to separate the whole blood to remove undesired elements such as red and white blood cells from the plasma, which contain antibodies and other proteins that are shed by viruses or bacteria [26]. The second component is the conjugate pad, where conjugate antibodies labelled with biorecognition elements (reporter particles) are immobilised. When the sample reaches the conjugate pad, the conjugated antibodies bind to the target analytes. The antibody–target complex then flows through the nitrocellulose membrane (via capillary force) to where the reaction happens on the test and control lines. The test and control lines comprise immobilised antibodies or proteins (depending on the type of target analytes) that will bind with the antibody–target complex and produce a signal attributed to the reporter particles. The remaining fluid is absorbed by the adsorbent pad (also known as the wicking pad), which is designed to capture the remaining sample and avoid backflow. The Schematic of LFIA is shown in Figure 1.

Once the sample has reached the test line on the membrane, the signal that appears on the test line can be visualised and interpreted to identify the presence of the analyte. The signal strength on the test line correlates with the number of targets that bind to the detector conjugate. Colloidal gold nanoparticles (AuNPs) have been utilised as reporter particles in LFIAs because of the vibrant colours emitted by their interaction with visible light. The optical properties of AuNPs are tunable by changing the size. For small (~30 nm) monodisperse AuNPs, the surface plasmon resonance phenomenon causes absorption of light in the blue–green portion of the spectrum (~450 nm) while red light (~700 nm) is reflected, producing a rich red colour. As the AuNPs size increases (~100 nm), a longer wavelength of surface plasmon resonance is absorbed, producing solutions with a pale blue or purple colour. The standard size of gold nanoparticles that have been used for LFIA is 40 nm.

The two main configurations of LFIA are primarily divided into the sandwich and competitive formats. The sandwich format is used to detect large targets that have at least two epitopes (binding sites). In contrast, the competitive format is used to detect small targets with a single epitope [27].

### 2.1. Sandwich Immunoassay

Sandwich LFIAs have been widely used for the detection of various disease biomarkers as well as small molecules, such as vitamin D [28]. Three different antibodies are usually used in this format [29]. (i) Conjugate antibodies recognise one of the target analyte’s epitopes, and they are immobilised on a conjugation pad linked to the reporter particles. When the sample is added, the conjugate antibody rehydrates and migrates to the test and control lines via capillary force. (ii) Capture antibodies are immobilised on the nitrocellulose membrane at the test line and are specific to another epitope of the target analyte. (iii) Species-specific anti-immunoglobulin antibodies that are immobilised on the control line membrane interact with the reaction antibody.

**Figure 2:**
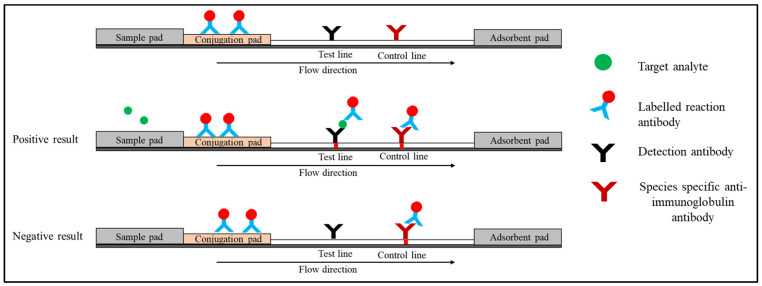
Schematic diagram shows the sandwich format of LFI.

When the specimen is applied to the sample pad, the target analyte binds with the conjugate antibody on the conjugate pad (analyte–Ab complex) and it is directed to the nitrocellulose membrane. This complex reacts with detection antibodies on the test line, resulting in the sandwich shown in Figure 2. On the control line, excess reaction antibody reacts with species-specific anti-immunoglobulin antibodies. Two red lines will appear at the test and control lines, indicating the presence of the target analyte [30]. In the absence of a target analyte, the reaction antibody only reacts with the species-specific anti-immunoglobulin antibody on the control line. At the control line, only one red line will appear. The presence of a control line indicates that the flow is completed and the test is valid. There are various strategies have been employed at the control line. The most popular approach is by utilising species-specific anti-IgG (Figure 2). Alternatively, researchers have also used an independent antibody–antigen complex such as biotinylated antibodies or oligonucleotides and bovine serum albumin-biotin conjugate [31,32,33].

### 2.2. Competitive Immunoassay (or Inhibition Immunoassay)

**Figure 3:**
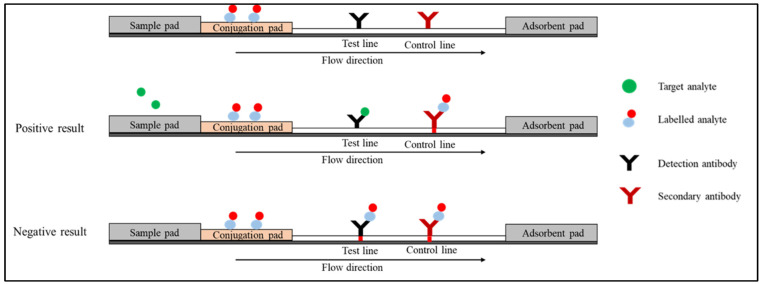
Schematic diagram shows the competitive format (first arrangement).

The competitive immunoassay format, also known as the inhibition immunoassay, can be conducted in two types of arrangements. In the first arrangement (Figure 3), the labelled analyte is attached to the conjugation pad. In the absence of the target analyte in the specimen, the labelled analyte moves through the strip bind detection antibody on the test line and the secondary antibody on the control line. The red colour will appear on both the test and control lines. In the presence of the target analyte, the unlabelled analyte in the specimen competes with the labelled analyte and binds to the test line. In the control line, the labelled analyte binds to the secondary antibody. Only a red line is formed on the control line [34].

**Figure 4:**
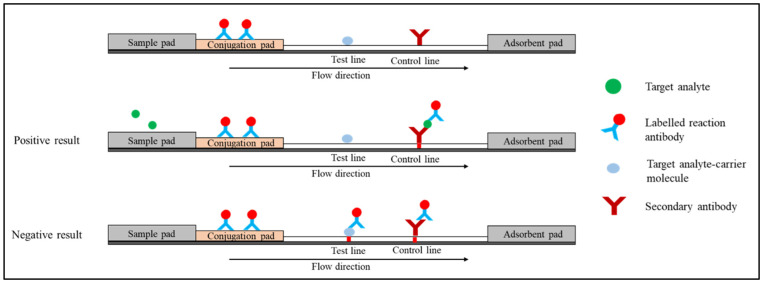
Schematic diagram shows competitive format (second arrangement).

In another arrangement (Figure 4), the conjugate pad is immobilised with a labelled reaction antibody. Target analyte-carrier molecule conjugate and secondary antibody are used in the test and control lines, respectively. The target analyte in the sample and the target analyte-carrier molecule on the test line compete for binding to the labelled reaction antibody. In the absence of the target analyte, the labelled reaction antibody moves through the strip and binds to the test line’s target analyte-carrier molecule conjugate and the control line’s secondary antibody. The red colour will appear on both test and control lines. In the presence of the target analyte, the labelled reaction antibodies react with the analyte in the sample and move to the control line. One red line is monitored at the control line on the strip [35].

## 3. Quantum Dot-Based Lateral Flow Immunoassay

**Figure 5:**
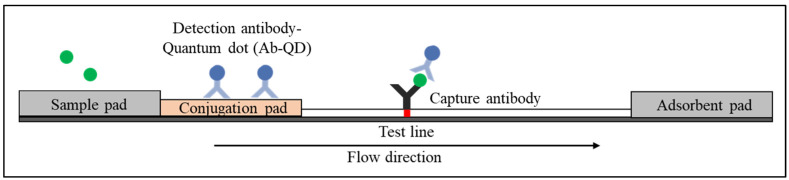
The configuration of the sandwich QD-based LFIA method.

Although gold nanoparticles (AuNPs) are widely used to label the conjugate probe, fluorescent reporters have attracted significant interest as they improve the sensitivity and detection limits of lateral flow immunoassays (LFIAs) [36,37]. Quantum dots (QDs) are the most auspicious fluorescent reporters due to their unique properties, which include high stability, high extinction coefficients, high quantum yields, and a long fluorescence lifespan [38,39]. These characteristics make QDs excellent reporters that can be functionalised with conjugate antibodies for the development of highly sensitive LFIAs. A schematic illustration of the QDs functionalised with conjugate antibodies in sandwich LFIAs is illustrated in Figure 5. The use of QDs as a reporter in sandwich LFIAs requires two antibodies wherein both the conjugate and capture antibodies are specific to the target of interest. Conjugate antibodies labelled with QDs are immobilised on the conjugate pad to enable measurable fluorescence detection. Meanwhile, on a nitrocellulose membrane, capture antibodies are immobilised to capture the target of interest, forming the QD-labelled antibody–target–antibody complex. This complex produces a bright fluorescent band in response to ultraviolet excitation. Similarly, QDs can also be used in competitive LFIAs format by functionalising the nanoparticles with reaction antibodies or analytes on the conjugate pad.

### 3.1. Types of Quantum Dots

Quantum dots (QDs) can be grouped into different types based on their components and structure. There are three main types of QDs, which include core-type QDs, core-shell QDs, and alloyed QDs.

#### 3.1.1. Core-Type Quantum Dots

Core-type QDs are single component nanoparticles with unvarying internal compositions, such as chalcogenides (selenides, sulfides, or tellurides) of metals such as cadmium, lead, or zinc. The examples include cadmium telluride (CdTe) [40] and lead sulfide (PbS) [41]. The core-type QDs’ electroluminescence and fluorescence properties can be fine-tuned by simply manipulating the crystallite size.

#### 3.1.2. Core–Shell Quantum Dots

The luminescent characteristics of QDs result from electron-hole pair recombination (exciton decay) via radiative pathways. However, exciton decay can also take place nonradiatively, lowering the fluorescence quantum yield. Growing shells of another higher band gap semiconducting nanocrystals around QDs is one method employed to enhance their efficiency and intensity [42]. Core–shell quantum dots (CSQDs) are nanocrystals with tiny regions of one nanocrystal embedded in another with a larger band gap (CSSNCs). QDs with cadmium selenide (CdSe) in the core and zinc selenide (ZnS) in the shell (CdSe/ZnS) have been used for sensitive detection of gastric cancer [43] and aflatoxin B1 [44]. Coating QDs with shells increases quantum yield by allowing nonradiative recombination sites to pass through, as well as making them more robust.

#### 3.1.3. Alloyed Quantum Dots

The ability to tune the optical and electronic properties by varying the crystallite size has become a distinguishing feature of QDs. Nonetheless, changing the crystallite size to tune the properties may cause problems in many applications with size constraints. Multicomponent dots provide an alternative method for tuning the properties without changing the size of the crystallite. The optical and electronic properties of alloyed semiconductor nanodots with both homogeneous and gradient internal structures can be tuned by simply altering the composition and internal structure without modifying the crystallite size. For example, alloyed QDs of the compositions CdSeS/ZnS with a diameter of 6nm emit light of varying wavelengths by simply altering the composition [45]. Alloyed semiconductor QDs, which were created by alloying two nanocrystals with distinct band gap energies, displayed unique optical characteristics.

### 3.2. Strategies for Conjugating Quantum Dot with Antibodies

The process of conjugating antibodies to the surface of QDs can be performed using several methods. Passive adsorption is the established technique for conjugating antibodies to the surface of QDs and is still widely used. Utilising the interactions (forces) between molecules and surfaces at a certain pH (e.g., van der Waals and ionic forces), antibodies can be directed to spontaneously bind to QDs to form a conjugate. The antibody is normally added in excess to certify that the entire surface of the QDs is covered. After the conjugation is finished, any antibody that is still free in the solution is removed using centrifugation or filtration.

Covalent binding is the most commonly used method for conjugating antibodies to the QDs surface. Covalent binding of QD to conjugate antibodies offers several advantages. Fewer numbers of antibodies are needed to increase the sensitivity, thus lowering the overall cost. Furthermore, covalent conjugates exhibit high stability, allowing their use in difficult sample matrices and high-salt buffering solutions. Additionally, conjugates are easily prepared without the need for extensive salt or pH optimisations, thus, meaning antibody screening experiments can be performed faster.

One common strategy for employing covalent binding is by functionalising the QD surfaces with carboxyl (carboxylic acid). However, obtaining QDs functionalised with carboxyl groups could be tricky. There are several methods to synthesise carboxyl-functionalised QDs have been previously reported. Mansur et al. [46] used acid-functionalised poly (vinyl alcohol) (PVA–COOH) polymer as a capping ligand to synthesise CdSe nanoparticles. The synthesis method was performed using the colloidal chemistry technique via the aqueous route at room temperature. Alternatively, Chen et al. [47] dissolved QDs in deionised water using an ultrasonic bath. Then, HCl solution was added to the solution and mechanically stirred to replace Na+ ions with H+, producing carboxyl-functionalised QD. Once the QDs functionalised with carboxyl groups, the carboxylic groups on QD surfaces can be linked to a primary amine in lysine residues of antibody using 1-Ethyl-3-(3-dimethylaminopropyl) carbodiimide (EDC) and N-hydroxysulfosuccinimide (NHS) reagents to form amide bonds [48,49]. Another approach is via reductive amination, by oxidising the oligosaccharides on the Fc region of the antibody followed by coupling to the amine- or hydrazide-functionalised QDs [50].

### 3.3. Performance of Quantum Dot-Based Lateral Flow Immunoassay

A literature search was conducted in April 2022 through PubMed using keywords combined with Boolean operators (Quantum dots AND Lateral Flow). After screening the search results, the final eight studies that involve the development of QD-LFIA for infectious diseases were reviewed. In this section, we summarised the strategies employed and published the performance of the QD-LFIA devices. Various QD-LFIAs have now been developed as point-of-care testing (POCT) for the surveillance of infectious diseases such as tuberculosis, Influenza A, Influenza B, tetanus, syphilis, and COVID-19 (Table 1).

Syphilis is a sexually transmitted infection (STI) that causes serious health problems worldwide. In 2010, Yang et al. [56] developed a QD-LFIA to be used for the screening of syphilis. The QD-LFIA was designed to detect anti-TP47 polyclonal antibodies by visualising the emission of CdTe under a portable ultraviolet lamp. Thioglycolic acid (TGA) was used to link the QDs with *Staphylococcal* Protein A (SPA). In the presence of anti-TP47 polyclonal antibodies, the QD-labelled SPA will form a complex with the antibodies. Then, the anti-TP47 antibodies will bind to the TP47 antigen immobilised on the test line, producing a signal that can be visualised under UV light. The assay is suitable for rapid indirect screening of syphilis as the turnaround time is only 10 min. With regard to the limit of detection, the QD-LFIA could detect as low as 2 ng/mL, which was tenfold higher than that of the AuNPs–based method. Yang et al. also evaluated the diagnostic performance of the assay using 50 *Treponema pallidum* particle agglutination (TPPA)-positive serum samples and 50 serum samples from a healthy volunteer. The evaluation showed 100% sensitivity and specificity, meaning that it is a promising replacement for the AuNPs–based method.

Our literature search found three studies that developed QD-LFIA for influenza viruses. In 2012, Li et al. [58] developed a novel QD-LFIA for rapid and highly sensitive detection of avian influenza virus subtype H5 in chicken serum samples. The LFIA utilised glutathione (GSH)-capped CdTe to improve the sensitivity of the immunoassay with a detection limit of 0.09 ng/mL. Analysis of the immunoassay using 20 clinical serum samples, which were collected from naturally infected chicken, showed a sensitivity of 100.0% and a specificity of 88.2%. In another study, QD-LFIA was developed to simultaneously detect influenza A virus subtypes H5 and H9 [55]. The antigen detection assay utilised carboxyl-functionalised CdSe/ZnS as a reporter, conjugated to influenza A virus subtype H5 and H9 antibodies via an amide bond. The QD-LFIA was able to rapidly (15 min) analyse the avian cloacal swab samples with a limit of detection of 0.016 HAU for influenza A virus subtypes H5 and 0.25 HAU for subtypes H9. In addition, the sensitivity and specificity analysis using RT-PCR as a reference test showed that the QD-LFIA demonstrated 100% accuracy. More recently, a study in Korea compared the performance of a newly developed QD-LFIA (QuantumPACK Easy; BioSquare Inc., Hwasung, Korea) with a commercially available LFIA (Sofia; Quidel, San Diego, CA, USA) for the detection of Influenza A and B viruses [52]. They evaluated the sensitivity and specificity of both assays using nasopharyngeal swab samples of 394 patients at the Asan Medical Center, Korea, confirmed with RT-qPCR as a reference test. The study found that the QuantumPACK Easy exhibited higher sensitivity of 80.9% (influenza A) and 83.7% (influenza B) than the Sofia assay (66.0% for influenza A and 61.2% for influenza B).

Tuberculosis, an infectious disease caused by the *Mycobacterium tuberculosis*, is a global public health problem and among the top 10 leading causes of death worldwide, particularly in low- and middle-income countries [59]. In an effort to prevent the spread of the disease, a QD-LFIA device based on a double-antibody sandwich format labelled with core–shell quantum dots (CdSe/ZnS) coupled with streptavidin was developed for the detection of *M. tuberculosis fprA* proteins [51]. The study reported that the device was able to detect *fprA* proteins in liquid samples at the lowest dilution of 12.5 pg/µL. The sensitivity of LFIA improved as compared to other immunochromatographic tests following the use of QDs labelled antibodies.

Owing to high target specificity and affinity, antibodies are considered to be the leading molecular recognition units in LFIAs [60]. Most of the available QD-LFIAs for the detection of infectious disease causative agents used whole antibody structures. Alternatively, the Fc fragments of antibodies can be used in indirect LFIA as a detector probe for the target antibodies. Taking advantage of this, a simpler QD-LFIA featuring goat anti-human IgG (Fc)-labelled QDs was successfully developed to detect tetanus antibodies in human serum [53]. Owing to the toxicity of cadmium, the researchers employed eco-friendly QDs (Cu:Zn−In−S/ZnS) as the reporter. The QD-LFIA showed a high sensitivity for tetanus antibody detection, with a detection limit 10 times lower than that of the AuNP-LFIA (0.001 IU/mL).

Following the declaration of the coronavirus disease 2019 (COVID-19) pandemic by the WHO, Wang et al. [54] reported a QD-LFIA that was used to concurrently detect SARS-CoV-2 spike (S) and nucleocapsid (N) antigens. They fabricated a magnetic quantum dot with a triple QD shell (MagTQD) into the LFIA device to produce superior fluorescence signals at 365 nm excitation of UV light. The monoclonal antibodies against the SARS-CoV-2 N antigen and S antigen were conjugated onto the carboxyl groups of the MagTQD surface by EDC/NHS coupling chemistry. The QD-LFIA showed a high sensitivity for both direct mode (1 pg/mL) and enrichment mode (0.5 pg/mL). In addition, the device is also efficient for rapid screening as the turnaround time from samples to results can be obtained within 10 min for the direct mode and an additional 20 min for the enrichment mode.

As an alternative to antibodies, aptamers can be used as capture and detection probes. In 2014, Bruno et al. [57] developed an aptamer-based lateral flow device for the detection of *Escherichia coli*. DNA aptamer of 60 nucleotides linked to Qdot 655 (Thermo Fisher Scientific, Waltham, MA, USA) via the streptavidin–biotin interaction was used as a detection probe. The binding between the Qdot–aptamer conjugate and the target occurred in the presence of whole-cell bacteria. The formed complexes continued to migrate along the nitrocellulose membrane and were captured by an amino labelled aptamer (73 nucleotides) that was immobilised on the test line. The fluorescence signals emitted by the Qdot 655 under UV light were evaluated using *E. coli* diluted in PBS and showed highly sensitive detection with a detection limit of 300 bacterial cells. The study provides a proof of principle for aptamer-based lateral flow system and significant improvement in sensitivity of the assay due to the use of QD. Additionally, the use of aptamers to replace the antibodies in the lateral flow system is beneficial as aptamers are cheaper, more stable, and have minimal batch-to-batch variation compared to the antibodies [61].

## 4. Conclusions

The present review summarises the strategies employed for utilising QDs to improve the sensitivity of LFIA. The use of QDs as a reporter in LFIAs enhanced the sensitivity of the immunoassays. Currently, only one study successfully utilised QDs in aptamer-based lateral flow assay for the detection of *E. coli*. One important gap found in the present review is that only a limited number of studies for QD-LFIAs are currently available. In order to improve the performance and effectiveness of POCT, the roadmap on LFIAs development should adopt the detection of analytes via QDs, particularly for various infectious diseases such as typhoid, AIDS, melioidosis, etc., as part of the preparedness strategies to combat the future pandemic. With the emergence of new infectious diseases, the sensitivity of LFIAs must be further improved to maximise their potential as POCT. QDs seem to be a promising reporter to achieve such improvement.

## Figures and Tables

**Table 1:** Summary of quantum dot-based lateral flow immunoassay.

No.	Pathogens	Targets	Samples	No of Samples	Capture Probes	Type of QDs	Size of QDs	Origin of QDs	Performance	References
1	*Mycobacterium tuberculosis*	*FprA* antigens	*FprA* antigens diluted in PBS	NR	anti-*FprA* antibodies	CdSe/ZnS	15–20 nm	commercial	LoD of 12.5 pg/μL in less than 10 min	[51]
2	Influenza A and B	N antigens	Human nasopharyngeal swab	394	Influenza A and B antibodies	NR	NR	commercial	Sensitivity of 80.9% for influenza A and 83.7% for influenza B and 100% specificity	[52]
3	*Clostridium tetani*	Tetanus antibody	Human serum spiked with tetanus antibody	NR	Tetanus antigens	Cu:Zn−In−S/ZnS	NR	synthesized	LoD of 0.001 IU/mL in 30 min	[53]
4	SARS-CoV-2	S and N antigens	Human saliva and nasal swab spiked with SARS-CoV-2 S and N antigens	NR	Monoclonal antibodies against SARS-CoV-2 N antigens and S antigens	MagTQD	160 nm	synthesized	LoD of 1 pg/mL for direct mode and 0.5 pg/mL for enrichment mode in 10 min	[54]
5	Influenza A	N antigens	Avian cloacal swab	147	Influenza A virus subtype H5 and H9 antibodies	CdSe/ZnS	25 nm	synthesized	100% accuracy and LoD of 0.016 HAU for H5 and 0.25 HAU for H9 in 15 min	[55]
6	*Treponema pallidum*	anti-TP47 polyclonal antibodies	Serum of syphilis patients and healthy individuals	100	TP47 antigen	CdTe	3.5 nm	synthesized	100% sensitivity and 100% specificity in 10 min, LoD of 2 ng/mL	[56]
7	*Escherichia coli*	Whole cells	*E. coli* diluted in PBS	NR	DNA aptamers	Qdot	NR	commercial	LoD of 300 bacterial cells	[57]
8	Influenza A	Influenza A virus subtype H5 antigens	Chicken serum samples	20	Influenza A virus subtype H5 antibodies	CdTe	NR	synthesized	LoD of 0.09 ng/mL. Turnaround time in 10 min. 100% sensitivity and 88.2% specificity.	[58]

HAUs; hemagglutinating units, IU; international units, LoD; limit of detection, N; nucleoprotein, NR; not reported, PBS; phosphate buffered saline, S; spike.

## Data Availability

Not applicable.
